# DNA Damage, an Innocent Bystander in Atrial Fibrillation and Other Cardiovascular Diseases?

**DOI:** 10.3389/fcvm.2020.00067

**Published:** 2020-04-28

**Authors:** Kennedy S. Ramos, Bianca J. J. M. Brundel

**Affiliations:** ^1^Department of Physiology, Amsterdam UMC, Vrije Universiteit, Amsterdam Cardiovascular Sciences, Amsterdam, Netherlands; ^2^Department of Cardiology, Erasmus Medical Center, Rotterdam, Netherlands

**Keywords:** PARP, DNA damage, atrial fibrillation, peripartum cardiomyopathy, dilated cardiomyopathy, metabolism, lamin a/c, atrial cardiomyopathy

## Abstract

Atrial Fibrillation (AF) is the most common clinical tachyarrhythmia with a strong tendency to progress in time. AF is difficult to treat and therefore there is a great need to dissect root causes of AF with the ultimate goal to develop mechanism-based (drug) therapies. New findings related to mechanisms driving AF progression indicate a prime role for DNA damage-induced metabolic remodeling. A recent study uncovered that AF results in oxidative DNA damage and consequently excessive poly-ADP-ribose polymerase 1 (PARP1) activation and nicotinamide adenine dinucleotide (NAD^+^) depletion and finally atrial cardiomyocyte electrical and contractile dysfunction. This newly elucidated role of DNA damage in AF opens opportunities for novel therapeutic strategies. Recently developed PARP inhibitors, such as ABT-888 and olaparib, provide beneficial effects in limiting experimental AF, and are also found to limit atherosclerotic coronary artery disease and heart failure. Another therapeutic option to protect against AF is to replenish the NAD^+^ pool by supplementation with NAD^+^ or its precursors, such as nicotinamide and nicotinamide riboside. In this review, we describe the role of DNA damage-mediated metabolic remodeling in AF and other cardiovascular diseases, discuss novel druggable targets for AF and highlight future directions for clinical trials with drugs directed at PARP1-NAD^+^ pathway with the ultimate aim to preserve quality of life and to attenuate severe complications such as heart failure or stroke in patients with AF.

## State of the Art: AF Healthcare Problem

AF is the most common progressive cardiac rhythm disorder affecting 2–3% of the Western population (1). In addition, AF is associated with serious complications such as stroke, heart failure and increased mortality (1). Compared to age-matched males, women have a higher risk of AF-related complications, including mortality and stroke (2), probably related to higher prevalence of specific risk factors including hypertension, obesity, electrophysiological differences, and absence of cardioprotective estrogens after menopause (3).

Well-known environmentally-induced risk factors associated with AF include aging and other cardiovascular diseases, including coronary artery disease, and acquired valvular heart diseases. These degenerative pathologies clustered within “wear-and-tear” program present predominance among elderly population (4). On the other hand, in up to 20% of AF patients, AF develops at a young ageand in the absence of “wear-and-tear” risk factors and gross structural heart changes (5, 6). Here, AF may be related to a genetic mutation. AF families have been identified carrying mutations in cytoskeletal proteins, including lamin A/C (*LMNA*) (7) and desmin (8), which have been associated with dilated (DCM) and peripartum cardiomyopathy (PPCM) onset (9).

At present, treatment modalities for AF are only moderately effective and do not prevent AF progression from recurrent intermittent episodes (paroxysmal) to persistent and finally permanent AF. Although invasive ablation therapy is promising in early stage AF, up to 60% of persistent AF patients show AF recurrence within 1 year and require multiple, expensive procedures (10). Pharmacological therapy of AF, which originates from the 1960s and is directed at inhibition of ion-channels, does not prevent AF progression in 85% of patients and its usage is limited by potentially severe and life-threatening side-effects (11). The response of an individual patient to pharmacological therapy can often not be predicted and selection is therefore based on “trial-and-error”. Due to the lack of effective treatment modalities for AF, AF progresses in time and hence has a significant physical, psychological, societal, and economic impact. Therefore, there is an urgent unmet need to develop new (pharmaco)-therapeutic strategies directed at inhibition of mechanistic root causes of AF.

Recently published findings reveal important evidence for AF promotion due to dysmorphic nuclei-associated DNA damage, and subsequent activation of the DNA repair protein poly-ADP-ribose polymerase (PARP), especially PARP1. In turn, PARP1 activation results in the depletion of NAD^+^ levels in mitochondria, which causes oxidative stress, additional DNA damage, energy depletion and AF progression (12). In this article, we review the current understanding of DNA damage-PARP1-NAD^+^ axis in the pathogenesis of AF and cardiovascular diseases. Furthermore, we discuss novel druggable targets for AF and highlight future directions for clinical trials with drugs directed at DNA damage-PARP1-NAD^+^ axis with the ultimate aim to preserve quality of life and to attenuate severe complications such as heart failure or stroke in patients with AF.

## Role of PARP1 in Conserving Genetic and Functional Integrity of the Cardiomyocyte

Maintaining the correct genetic sequence is crucial for a healthy function of dividing but also for differentiated cells, such as cardiomyocytes. Due to environmentally-induced “wear-and-tear” or by design (genetics), alterations in the genetic sequence are induced. These alterations include single-strand breaks (SSBs) and double-strand breaks (DSBs) (13). As a response to these potential harmful DNA breaks, a complex machinery of DNA surveillance becomes activated to recognize the DNA damage, repair the breaks, or in case of excessive DNA damage, initiate the process of cell death (14).

**Figure 1:**
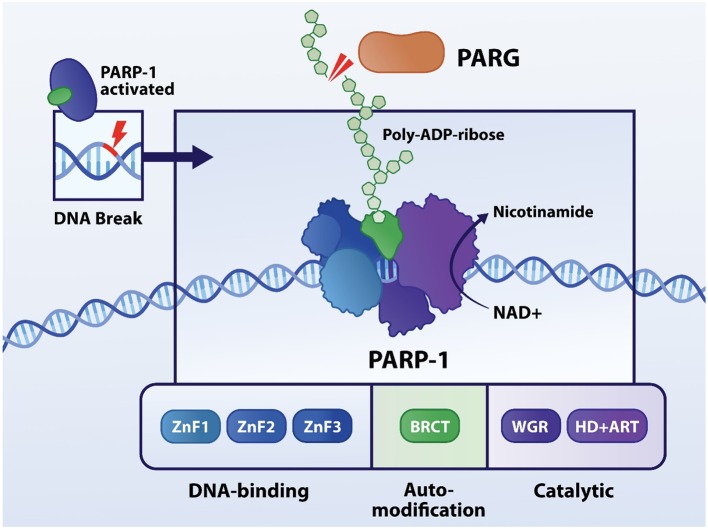
Structure and function of PARP1. PARP1 contains three functional domains: DNA-binding, auto-modification and catalytic domain. The three zinc fingers are responsible for recognizing aberrances in DNA molecule, followed by binding to it. In the center, the auto-modification domain permits PARylation of PARP itself. Finally, the catalytic domain carries the PARP signature and is responsible for transferring ADP-ribose subunits from nicotinamide adenine dinucleotide (NAD^+^) to PAR onto nuclear acceptor proteins.

PARP is a superfamily of six nuclear ADP-ribosyl transferase enzymes that are activated by SSBs and DSBs, serving to recruit the DNA repair machinery by synthesis of poly-ADP-ribose (PAR) chains. Poly-ADP-ribosylation (PARylation) is a post-translation modification of nuclear proteins guided by PARsynthesis. PARP1, as the most abundant nuclear family member, has three different functional domains. Firstly, zinc fingers are crucial to recognize SSBs and/or DSBs in DNA and bind to them (Figure 1). In the center, the auto-modification domain permits PARylation of PARP itself (15). Finally, the catalytic domain carries the PARP signature and is responsible for transferring ADP-ribose subunits from nicotinamide adenine dinucleotide (NAD^+^) to PAR and onto nuclear acceptor proteins. Simultaneously to PAR polymerization, PARP1's affinity to the damaged DNA site is weakened, due to the dense negative charge and size of the new polymer (16). Hence allowing recruitment and coupling of proteins which belong to the DNA repair machinery, including the DNA base excision repair (BER) machinery. Polymer growth is limited by poly-ADP-ribose glycohydrolase (PARG) that removes PAR from PARP1 by cleaving ribose-ribose bonds (17), allowing PARP1 to recognize other damaged DNA loci and initiate a new signaling and reparative cycle (18). Under healthy conditions PARP1 activity is low, however upon “wear-and-tear” and genetic conditions its catalytic activity can increase from 10 to 500-fold (19, 20). During excessive PARP activation high levels of PAR chains are synthesized. Hereto, NAD^+^ is consumed by PARP up to an extent that it depletes cellular NAD^+^ levels. NAD^+^ is a cofactor that is central in the metabolism of cells, including cardiomyocytes. NAD^+^ acts in redox reactions by carrying electrons from one reaction to another and as such, a reduction in mitochondrial NAD^+^ levels is associated with diminished capacity for ATP production (21). To restore the NAD^+^ pool, this co-enzyme is resynthesized. NAD^+^ resynthetization is an energy consuming process as specific steps in the glycolysis to produce ATP are NAD^+^ dependent. As such, NAD^+^ depletion leads to loss in overall ATP levels (22–24). This scenario of energy failure contributes to a metabolic type of cell death via PAR-induced translocation of apoptosis-inducing factor (AIF) from the mitochondria to nucleus (25).

In addition to PARP1-induced NAD^+^ and ATP depletion, research findings also revealed that NAD^+^ and ATP depletion results in a biochemical and physiological imbalance by excessive production of reactive oxidant species (ROS) by mitochondria. When not efficiently compensated by antioxidant reagents, endogenous ROS have a potential deleterious influence on cellular structures, including nuclear DNA and mitochondrial DNA molecules (26). An extensive body of work has recognized the importance of ROS as a mediator on DNA damage. ROS can directly cause oxidative damage on nucleotide bases (27), including bases which share peripheral loci. Consequently, oxidized bases are recognized and repaired by the BER machinery (28), particularly glycosylase mediated base excision is firstly induced. Likewise, when such a process is simultaneously demanded on damaged bases, belonging to opposite strands, the attempt of repair actually generates a DSBs (29), resulting in an enhancement of DNA damage, PARP1 activation, and NAD^+^ depletion.

In summary, oxidative stress may cause DNA damage and therefore activates especially PARP1, resulting in NAD^+^ depletion and consequently drive further oxidative protein and DNA damage. This feed-forward mechanism is found to be associated with several cardiovascular diseases, including AF. As such, the activation of the DNA damage-PARP1-NAD^+^ axis plays an important role in impairment of genomic integrity, mitochondria control, and ultimately result in cardiomyocyte dysfunction (19).

## Role of DNA Damage-PARP1-NAD^+^ Axis in Atrial Fibrillation

**Figure 2:**
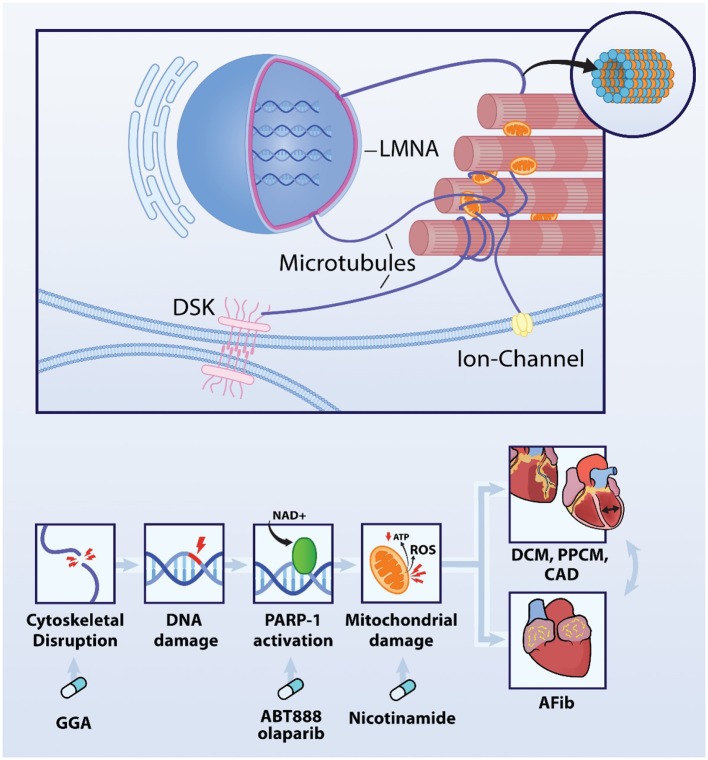
DNA damage-PARP1-NAD^+^ axis is involved in AF and additional cardiovascular diseases. AF causes disruption of the microtubule network, DNA damage, PARP1 activation and excessive depletion of the NAD^+^ levels resulting in ATP depletion and ROS production in the mitochondria. This mechanism is driving AF, Interestingly, comparable mechanism is obverved in *LMNA* mutation-induced DCM and PPCM as well as atherosclerotic CAD. Importantly, AF is often associated with these cardiovascular diseases, and vice versa indicating that mechanisms driving these cardiac diseases may the enhance each other. Key modulators within this axis are druggable targets as conservation of the cytoskeletal network with the HSP-inducer geranylgeranylacetone (GGA), PARP1 inhibitors ABT-888 and olaparib and precursor of NAD^+^, nicotinamide protect against AF, CAD, DCM, and PPCM.

Recently published findings reveal important evidence for the presence of dysmorphic nuclei which associate with DNA damage and increased PARP1 activation in experimental and clinical AF (12). In turn, PARP1 activation results in consumption of NAD^+^ levels from mitochondria to such an extent, that it depletes intracellular NAD^+^ levels, thereby exacerbating oxidative damage to proteins and DNA (Figure 2). Activation of this sequel is likely triggered by a substantial increase in myocardial energy demand resulting from the four- to six-fold increase in electrical and contractile activity during AF episodes. Subsequent failure to meet the increased energy demand results in progressive dysfunction of mitochondria, oxidative damage to proteins and DNA and has been associated with disruption of the microtubule network (Figure 2) (30). DNA damage then activates PARP1 initiating the depletion of NAD^+^. This feed-forward mechanism is precluded by replenishment of NAD^+^ levels and pharmacological inhibition of PARP1 with 3-aminobenzamide (3AB), veliparib (ABT-888), or olaparib and genetic depletion of PARP1 (30). Heat Shock Proteins (HSP) are crucial to ensure balanced protein synthesis, folding and clearance and also stabilization of the structural protein network (i.e., proteostasis) (31). This ensured proteostasis attenuates AF-induced microtubule network disruption and consequently ameliorates DNA damage-induced PARP1 activation and NAD^+^ depletion in atrial cardiomyocytes. Consistent with these findings, atrial cardiomyocytes of patients with persistent AF also show significant DNA damage, which correlates with PARP1 activity. In addition, in atrial cardiomyocytes, DNA damage was associated with electrophysiological deterioration, including prolongation of action potential duration (possibly via the reduction in potassium channel expression (32, 33), reduction in cardiomyocyte excitability and increased dispersion of action potential duration, thereby creating a molecular and structural substrate for further arrhythmogenesis In tachypaced atrial cardiomyocytes, PARP inhibitors prevent PARP1 activation and consequently NAD^+^ depletion, and thereby protect against electrophysiological deterioration, and arrhytmicity (12). These findings not only indicate a novel mechanism by which AF impairs atrial cardiomyocyte function, but also indicate PARP1-inhibition, NAD^+^ supplementation and/or microtubule conservation as a possible therapeutic target that may preserve atrial cardiomyocyte function in clinical AF. Further studies are still necessary to ensure similar results in other cardiac cell types.

## Role of DNA Damage-PARP1-NAD^+^ Axis in Coronary Artery Disease

Emerging evidence indicates that hypoxia followed by re-oxygenation promotes oxidative stress, release of free radicals and oxidizing species which, in turn, promote DNA damage, activate PARP by stimulating PARylation, and ultimately result in depletion of NAD^+^ and ATP levels (Figure 2) (34, 35). In studies using rat and rabbit models of ischemia-reperfusion, administration of the PARP inhibitor 3AB resulted in an improvement in heart function, including systolic and diastolic function, and reduced the size of ischemic areas in left ventricular wall compared to non-treated control animals, who showed elevated levels of necrosis, neutrophil infiltration and reduced levels of ATP (35, 36). In addition, findings from hypoxia-reoxygenation *in vitro* models of cardiomyoblasts, also revealed PARP activation, energy loss, exuberant necrosis, and AIF dependent apoptosis. Moreover, administration of the PARP inhibitor PJ34 prevented apoptosis (34). The role of PARP as modulator of the pathway of cell death, triggered by ischemic/infarction conditions, has also been investigated in rat models. Here, administration of the PARP inhibitor PJ34 promoted a shift from an expected major necrosis, which is pro-inflammatory driven, toward a less harmful and programmed apoptosis process intermediated by AIF (37). In this model, initial damage is not massively avoided, instead the chronic tissue damage due to energy metabolites, exuberant inflammation and cell death induction, can be diminished toward less harmful standards (38, 39). Thus, the findings to date indicate that the DNA damage-PARP1-NAD^+^ axis is also indicated in coronary artery disease and therefore may represent a novel target for therapeutic treatment.

## DNA Damage-PARP1-NAD^+^ Axis in *LMNA* Mutation-Induced Dilated Cardiomyopathy

Dilated cardiomyopathy (DCM) is a clinical diagnosis characterized by left or bi-ventricular enlargement which implies inefficient systolic function, and ultimately results in heart failure or sudden cardiac arrest (40). Interestingly, 40% of DCM cases rely their etiology on genetic mutations (41). Among the DCM-related mutant genes, mutations in cytoskeletal protein *LMNA* have a high prevalence (41). Interestingly, several AF families have been identified that carry a mutation in *LMNA*, suggesting that this mutation also is involved in AF onset (7). The intermediate filament protein LMNA is linked via microtubule network with the outer cell membrane, with sarcomeric proteins such as titin, Z-disk and the nuclear membrane, and thereby regulates sarcomere architecture and function but also nuclear morphology, DNA stability and thereby gene expression (Figure 2) (42–45). Malfunction of LMNA proteins has been associated with cardiac manifestations such as compromised conduction disorders, and arrhythmogenesis, and consequently contributes to clinical phenotype compatible with DCM (46). So far, studies in ventricular cardiomyocytes revealed that *LMNA* mutations result in cytoskeletal and microtubule disruption (44, 47), dysmorphology of the nuclei (42), activation of the DNA damage response (48) and PARP1 activation (49, 50) followed by consumption of mitochondrial NAD^+^ levels, which drive cardiomyocyte dysfunction and cardiomyopathy onset (49, 51). In DCM, all these effects were ameliorated by supplementation with a precursor of NAD^+^, nicotinamide (50), or conservation of the cytoskeletal network with GGA, a HSP-inducer (47).

Thus, the findings indicate that mutations in cytoskeletal protein *LMNA* result in DNA damage, PARP1 activation and NAD^+^ depletion in ventricular cardiomyocytes. It is still not entirely understood whether the same mechanism underlies cytoskeletal protein mutation in atrial cardiomyocytes, thereby potentially inducing AF in *LMNA* mutation carriers.

## DNA Damage on Peripartum Cardiomyopathy

Peripartum Cardiomyopathy (PPCM) is a dilated cardiomyopathy which manifests in the last trimester of the pregnancy, during delivery or during the first six months postpartum (52). Its diagnosis is made by exclusion criteria: when the left ventricle ejection faction is below 45%, unexplained by any other underlying cardiovascular disease, during the mentioned period (53). In accordance with other cardiovascular diseases, its incidence has progressively increased over the course of recent years. Recent studies have brought the etiology of PPCM toward a multi factor syndrome, likewise several pathophysiological mechanisms may underlie PPCM induction and/or progression. PPCM in combination with AF accelerates disease progression and impairs recovery from PPCM, indicating AF as an enhancer of PPCM severity (54). As a major potential common pathway, excessive oxidative stress associated with impaired antioxidant compensation seems to drive the development of PPCM phenotype (55). As part of the physiological changes during pregnancy (56), there is a higher oxidative stress, consequently enhancing ROS production. This pro-oxidative state seems to play an important role promoting DNA damage response pathway (57). Interestingly, recent research findings indicate a link between the expression of pathological gene variants of cardiomyopathy and DNA damage response pathway and PPCM. Whole exome sequencing in PPCM patients revealed that in addition to an increase in pathological cardiomyopathy gene variants, such as *LMNA* mutation, also an increase in gene variants affecting the DNA damage response pathway are associated with PPCM (Figure 2). These observations clearly point to a role for DNA damage pathway as key modulator in PPCM as well as *LMNA* mutation-induced PPCM. Future studies should reveal the exact role of DNA damage-PARP1-NAD^+^ axis in PPCM and AF associated PPCM.

## Intervention at the DNA Damage-PARP1-NAD^+^ Axis as a Novel Therapeutic Approach of AF

The identification of the prime role of DNA damage-PARP1-NAD^+^ axis in AF, liaises a wide variety of potential novel therapeutic approaches in AF. In order to prevent AF-induced cardiomyocyte dysfunction and potential AF progression, several targets for drug treatment that have been studied under experimental conditions, have shown promising findings and therefore may be applicable to the clinical field. These drugs include PARP inhibitors, NAD^+^ replenishment with nicotinamide and HSP-inducing compounds such as GGA (Table 1). An extensive body of work has been dedicated to develop optimal PARP inhibitors and to improve pharmacokinetics-dynamics, with the aim to select a drug with improved specificity, cardiac delivery and less drug-drug interaction. Even though the first PARP inhibitors were described 40 years ago (61), an exuberant increase in clinical trials has been reported recently. For example, the PARP1 inhibitor olaparib is already in phase III clinical trials for the treatment of metastatic breast cancer (62) and ABT-888 in phase I/II clinical trials for the treatment of ovarian cancers presenting BRCA mutations (Table 1). Olaparib is a favorable drug as it presents with high potency, low toxicity, and limited influence on QT/QTc interval (63). The second promising therapeutic strategy to prevent cardiomyocyte malfunction due to energetic failure is exogenous replenishment of NAD^+^ by its precursors nicotinamide riboside and nicotinamide (12, 30). Nicotinamide can be converted into NAD^+^ via the savage pathway. Also, nicotinamide acts as a PARP inhibitor claiming a synergetic dual pharmacodynamic effect. As deprivation of cardiac energy is associated with heart failure (64), mice treated with nicotinamide show amelioration in left ventricular contractile dysfunction and chamber dilatation (65), and consequently reveal attenuation of heart failure progression (49). The high translational potential and applicability in humans has been recently shown in an open-label pharmacokinetics study with nicotinamide ribose (niagen®, Chromadex) in healthy subjects. Here, niagen showed good up-take tolerance (even up to 2x 1000 mg/day) and resulted in an increase in circulating NAD^+^ levels (66). As AF is also associated with PARP1-induced NAD^+^ deprivation, replenishment of the intracellular NAD^+^ pools with nicotinamide riboside may represent a potential novel therapy in AF. Also, HSP-inducing drugs, such as geranylgeranylacetone (GGA) and GGA derivatives, may represent interesting drugs to treat AF patients. GGA and GGA derivatives were found to attenuate tachypacing-induced electrical and contractile dysfunction by conserving the microtubule network in atrial cardiomyocyte, *Drosophila* and dog models for AF (58, 67, 68). HSP-inducing compounds may also attenuate DNA damage-induced PARP1 activation and NAD^+^ depletion in AF, as GGA was previously observed to attenuate this pathway in *LMNA* mutant-induced DCM (47). We are advocating for clinical studies to establish whether these marketed and mechanism-based drugs are able to reduce the burden of AF and AF-related complications such as stroke and heart failure.

**Table 1:** Drugs targeting DNA damage-PARP1-NAD^+^ axis with potential benefit in AF.

**Key modulators**	**Drug**	**Action**	**Clinical phase**	**Indication**	**Ref/identifier (clinicaltrails.gov)**
I. Direct targeting DNA damage-PARP1-NAD^+^ axis					
DNA damage	Veliparib	PARP inhibitor	Phase II Phase II Phase I Phase II Phase II Preclinical	Metastatic breast cancer Hepatocellular carcinoma Adult solid neoplasm Ovarian cancer Colorectal cancer AF	NCT01009788 NCT01205828 NCT01154426 NCT01113957 NCT01051596 (12)
DNA damage	Olaparib	PARP inhibitor	Phase I Phase III Phase III	Ovarian cancer Pancreatic cancer Fallopian Tube Clear Cell Adenocarcinoma	NCT01237067 NCT02184195 NCT02446600
Mitochondrial dysfunction	Nicotinamide (niagen®)	PARP inhibitor Sirt inhibitor NAD-precursor	Phase III Phase III Phase II Phase II Preclinical	Lung carcinoma Chronic kidney disease Neurodegenerative disease Alzheimer's disease AF	NCT02416739 NCT02258074 NCT01589809 NCT00580931 (30)
II. Indirect targeting conservation of cytoskeleton cardiomyocytes					
HSP	GGA (teprenone)	HSP induction	Phase IV Phase IV Phase IV Preclinical Phase II	Gastric ulcers Gastritis Gastric lesion AF Cardiac bypass surgery	NCT01190657 NCT015475559 NCT01397448 (58, 59)
HSP	GGA derivatives	HSP induction	Preclinical	AF	(60)

## Summary

Atrial Fibrillation is the most common cardiac arrhythmia with a strong tendency to progress in time. Emerging evidence on mechanisms driving AF progression indicate a prime role for DNA damage-induced metabolic remodeling in atrial cardiomyocytes. This newly elucidated role of DNA damage in AF opens opportunities for novel therapeutic strategies, including PARP inhibitors, nicotinamide and nicotinamide riboside and HSP-inducing compounds. In addition, a comparable role for DNA damage has been observed in other cardiac diseases, including *LMNA* mutation-induced dilated cardiomyopathy, peripartum cardiomyopathy and coronary artery disease. This observation indicates a bidirectional influence between AF and these other cardiovascular diseases, linked by a general DNA damage-induced metabolic remodeling pathway. As such the DNA damage-induced metabolic remodeling pathway underlies atrial as well as ventricular cardiomyopathy.

## Author Contributions

KR and BB discussed the content, drafted and finalized the manuscript, and designed the figures.

## Conflict of Interest

The authors declare that the research was conducted in the absence of any commercial or financial relationships that could be construed as a potential conflict of interest.
